# Matched oligoclonal bands: Diagnostic utility and clinical characteristics

**DOI:** 10.1002/acn3.52162

**Published:** 2024-10-22

**Authors:** Yoji Hoshina, Justin R. Abbatemarco, Stefanie J. Rodenbeck, Jason T. Poon, Suzanne C. Liu, M. Mateo Paz Soldan, John E. Greenlee, John W. Rose, Lisa K. Peterson, Lisa Johnson, Alen Delic, Tammy L. Smith, Stacey L. Clardy

**Affiliations:** ^1^ Department of Neurology University of Utah Salt Lake City Utah USA; ^2^ Mellen Center for Multiple Sclerosis Cleveland Clinic Foundation Cleveland Ohio USA; ^3^ Department of Neurology Indiana University School of Medicine Indianapolis Indiana USA; ^4^ Department of Neurosciences Evergreen Health Kirkland WA USA; ^5^ Pediatric Neurology Division, Department of Pediatrics University of Utah Salt Lake City Utah USA; ^6^ George E. Wahlen Veterans Affairs Medical Center Salt Lake City Utah USA; ^7^ ARUP Institute for Clinical and Experimental Pathology Salt Lake City Utah USA; ^8^ Department of Pathology University of Utah Salt Lake City Utah USA; ^9^ Department of Laboratory Medicine and Pathology University of Washington Seattle WA USA

## Abstract

**Objective:**

To describe patient clinical characteristics associated with matched oligoclonal bands (OCB).

**Methods:**

A retrospective review at the University of Utah examined patients with matched OCB from 2015 to 2020. Clinical data, diagnosis, and outcomes were collected. Patients were classified with either multiple sclerosis (MS), other inflammatory neurologic disorder (other‐IND), or noninflammatory neurologic disorder (NIND).

**Results:**

Of 539 identified patients, 436 (53.4% female) were matched‐only, while 103 (43.7% female) were matched + unique. Patients with matched‐only bands were older (57.4 ± 16 vs. 52 ± 14.2, *p* < 0.001) and more likely to have a history of autoimmune disease (40.1% vs. 28.2%, *p* = 0.024) and/or cancer (28.7% vs. 16.5%, *p* = 0.012). Patients with matched + unique bands were more likely to have CSF pleocytosis (52.4% vs. 25.9%, *p* < 0.001), high IgG index (52.2% vs. 7.6%, *p* < 0.001), and an abnormal MRI (86.9% vs. 63.1%, *p* < 0.001). More than two‐thirds of matched‐only patients had NIND, while 33% and 41.7% of matched + unique patients had MS and other‐IND, respectively. Patients exhibiting matched‐only bands and a high IgG index demonstrated a significantly higher incidence of other‐IND compared to those with matched‐only bands and a normal IgG index (55.6% vs. 30.4%, *p* = 0.013). While Kaplan–Meier survival curves demonstrated higher mortality in the matched‐only cohort compared to the matched + unique cohort (*p* = 0.02), multivariable Cox regression analysis showed this difference was not statistically significant when adjusting for various factors. A history of cancer was the significant predictor of increased mortality risk (Hazard ratio = 3.147, 95% CI [2.196, 4.51]).

**Interpretation:**

Patients with matched only versus matched + unique OCB have distinct clinical profiles.

## Introduction

The detection of unique oligoclonal bands (OCB) in the cerebrospinal fluid (CSF) has long been used to support the diagnosis of multiple sclerosis (MS), as evidenced by inclusion of unique OCB in the 2017 McDonald Criteria.^1^ Although less common, other conditions including central nervous system (CNS) infection, autoimmune encephalitis, and even noninflammatory neurologic diseases can also be associated with unique OCB.^2^

From a laboratory perspective, OCB found only in the CSF and *not* in paired serum sample are considered unique OCB. This finding of unique OCB supports intrathecal synthesis of immunoglobulin G (IgG) and is generally considered clinically significant when at least two bands are identified through isoelectric focusing.^3^ Historically, identical OCB detected both in serum and CSF have been termed matched (or paired) OCB and have been considered to represent penetration of a systemic immune response across the blood–brain barrier. Paired OCBs may be detected in conditions such as Sjögren's syndrome, systemic lupus erythematosus, sarcoidosis, or malignancy.^4^, ^5^ The concurrent detection of both unique and matched OCB (matched + unique OCB) may indicate either CNS intrathecal IgG synthesis secondary to systemic inflammation or represent two independent processes: systemic inflammation and discrete CNS IgG synthesis.^4^, ^5^

Much of the literature regarding OCB relates to their utility in the diagnosis of MS.^1^, ^3^ Given the rapidly evolving field of autoimmune neurology, OCB are increasingly tested outside the context of suspected MS. Many patients undergoing evaluation for suspected neuroimmunologic disease are ultimately found to have matched bands in both the CSF and serum without unique OCB (matched‐only) or both matched and unique OCB (matched + unique). The significance of these laboratory findings and the clinical characteristics of these patients have not been closely examined. The objective of this study was to characterize the clinical features of patients with matched bands and determine the diagnostic utility of this laboratory finding.

## Methods

This was a retrospective observational study that followed the Strengthening the Reporting of Observational Studies in Epidemiology (STROBE) reporting guideline for observational studies conducted at University of Utah Health, Salt Lake City, Utah. Protocols (#00108537 and #00137511) were reviewed and approved by the University of Utah institutional review board and were determined to be exempt from further review.

Clinical data obtained during routine care for all adult patients (≥18 years old) evaluated at University of Utah Health between 1 January 2015 and 24 September 2020, who had OCB analysis as a component of a CSF examination, were collected through retrospective chart review. OCB patterns were classified into five distinct types based on their electrophoretic profiles. Type 1 indicates that no OCB are detected in both CSF and serum. Type 2 is characterized by the presence of at least two CSF‐restricted bands but not in the serum, indicating intrathecal IgG synthesis. Type 3 involves the presence of OCB in both CSF and serum, with additional at least two CSF‐restricted bands, suggesting both systemic and intrathecal IgG synthesis. Type 4 denotes identical OCB in both CSF and serum, suggesting a systemic immune response with blood–brain barrier penetration. Type 5 is defined by monoclonal bands that are identical in both CSF and serum, indicative of a monoclonal gammopathy. Only patients with Type 3 (matched + unique) and Type 4 (matched‐only) OCB patterns, who were also seen in‐person at University of Utah Health with available clinical data, were included in the analysis.

Demographic and clinical data, history of autoimmune disorders and cancer, serum protein electrophoresis (SPEP) and immunofixation, and neuroaxis MRI data at the time of CSF examination were extracted from the medical record. Neuroaxis MRI data were classified as normal or abnormal based on radiology reports and interpreted by autoimmune neurology‐trained neurologists (JRA, SJR, and JTP). The abnormalities were defined in the context of clinically significant findings, distinguishing pathological changes from incidental or age‐related variations. CSF cell count, protein, glucose, and IgG index results were also collected. CSF white blood cell (WBC) count >5 cells/μL, CSF total protein >45 mg/dL, and CSF glucose <50 mg/dL or <2/3 of serum glucose were defined as abnormal. IgG index elevation was defined as >0.66. These laboratory data abnormalities were defined according to the ARUP Laboratories reference intervals. Patients who had CSF red blood cell (RBC) ≥500 cells/μL were considered a traumatic tap and excluded from the study to avoid false elevation of WBC and protein. Patients' final diagnosis, which prompted the initial CSF evaluation, was classified into the following categories based on previously described criteria with minor modification: (1) MS, (2) other inflammatory neurological disorder (other‐IND), and (3) noninflammatory neurological disorder (NIND).^6^ More detailed classification information for each category is described in Supplement 1. A subanalysis comparing the final diagnosis in the matched‐only group with normal and elevated IgG index was also conducted. In these groups, the albumin quotient (QAlb) was verified to evaluate brain‐CSF barrier dysfunction based on an age‐dependent standard value, using the formula QAlb = [4 + (age/15)] × 10^−3^, as previously reported.^7^

Detection of OCB was performed by isoelectric focusing followed by immunofixation for IgG using a commercially available kit (HYDRAGEL 9 CSF Isofocusing kit, Sebia, Norcross, GA) on a semi‐automated HYDRASYS system. Briefly, paired serum and CSF samples were run side‐by‐side on an agarose gel. Immunofixation with enzyme‐labeled antisera to IgG was used to detect clones.^8^ The results of OCB patterns were interpreted by an experienced clinical laboratory technologist blinded to patient information. If results were equivocal, the run was repeated or reinterpreted by a second experienced technologist to reach a consensus.

All laboratory analyses were carried out at ARUP Laboratories (Salt Lake City, UT), and working methods were evaluated by internal and external quality management. Control measurements were carried out according to the manufacturer's guidelines. International consensus statements were followed on the processing and analysis of CSF.^9^

Patient demographics, medical history, and MRI features were summarized with descriptive statistics and stratified by banding pattern. Continuous variables are presented as mean ± standard deviation or median with interquartile range, as appropriate for their distribution. Categorical variables were shown as frequencies (percentages). Student's *t*‐test and Mann–Whitney U test were used to assess for significant differences for continuous variables, as appropriate for their distribution. The chi‐square and Fisher's exact tests were used for qualitative variables. Kaplan–Meier survival curves were used to estimate survival probabilities, and differences between groups were assessed using the log‐rank test. To assess long‐term outcomes and conduct a comprehensive survival analysis, follow‐up data were extended beyond the initial study period. The last follow‐up date for the survival outcomes was 30 November 2023. Cox proportional hazard models were employed to adjust for potential confounders and to identify factors associated with survival outcomes. Hazard ratios (HR) and 95% confidence intervals were reported. Statistical significance was set at *p* < 0.05. Analysis was conducted using R (version 4.3.2; R Foundation).

## Results

In total, 619 patients met the search criteria; 9 were excluded owing to duplicate data, 6 owing to incomplete data, and 65 owing to CSF RBC ≥500 cells/μL (Supplement 2), leaving 539 patients for further detailed analysis. Table 1 summarizes clinical features, laboratory and imaging results, and the outcomes of the cohort. A total of 436 patients (53.4% female) had matched OCB in the serum and CSF (matched‐only), while 103 patients (43.7% female) had both matched and unique OCB (matched + unique). Patients with matched‐only were older compared to matched + unique (57.4 ± 16 vs. 52 ± 14.2, *p* < 0.001). Within the entire cohort, 37.8% (*n* = 204) of the patients had one or more previously known autoimmune diseases. Of patients with matched‐only OCB, 40.1% (*n* = 175) had previously diagnosed autoimmune disease versus 28.2% (*n* = 29) of patients with matched + unique OCB (*p* = 0.024). In addition, patients with matched‐only were more likely to have a history of one or more cancers, compared to the matched + unique cohort (28.7% vs. 16.5%, *p* = 0.012), though there was no clear difference between the risk of either hematologic and nonhematologic malignancy in these groups. While the initial clinical symptoms prompting CSF evaluation were similar between the two cohorts, encephalopathy and weakness being common in both cohorts, sensory changes were more common in the matched + unique (18.4% vs. 8.5%, *p* = 0.003). Patients with matched + unique had more CSF pleocytosis (52.4% vs. 25.9%, *p* < 0.001), elevated IgG index (52.2% vs. 7.6%, *p* < 0.001), and neuroaxis MRI abnormalities (86.9% vs. 63.1%, *p* < 0.001). The median follow‐up period for matched‐only and matched + unique were 24 months and 39 months, respectively (*p* = 0.102).

**Table 1:** Demographics and clinical data.

	Matched OCB (*n* = 436)	Matched + unique OCB (*n* = 103)	*p*‐value
Age, years, mean [SD]	57.4 [16]	52.0 [14.2]	<0.001
Female, *n* (%)	233 (53.4)	45 (43.7)	0.075
History of autoimmune disorder, *n* (%)	175 (40.1)	29 (28.2)	0.024
Cancer, *n* (%)	125 (28.7)	17 (16.5)	0.012
Hematologic, *n* (%)	42 (9.6)	4 (3.9)	0.076
Lymphoma/leukemia, *n*	35	3	
Multiple myeloma, *n*	7	0	
Polycythemia vera, *n*	0	1	
Nonhematologic, *n* (%)	86 (19.7)	14 (13.6)	0.15
Small‐cell lung cancer, *n*	1	3	
Non‐small‐cell lung cancer, *n*	9	1	
Breast cancer, *n*	18	2	
Colon cancer, *n*	5	1	
Pancreatic cancer, *n*	2	0	
Thyroid cancer, *n*	1	1	
Renal cell carcinoma, *n*	12	1	
Prostate cancer, *n*	8	0	
Testicular cancer, *n*	2	0	
Ovarian cancer, *n*	3	1	
Melanoma, *n*	17	0	
Other, *n*	12^a^	5^b^	
Symptom prompting CSF testing			
Encephalopathy, *n* (%)	147 (33.7)	24 (23.3)	0.041
Weakness, *n* (%)	107 (24.5)	26 (25.2)	0.882
Seizure, *n* (%)	51 (11.7)	6 (5.8)	0.107
Sensory changes, *n* (%)	37 (8.5)	19 (18.4)	0.003
Gait instability, *n* (%)	21 (4.8)	9 (8.7)	0.148
Autonomic dysfunction, *n* (%)	6 (1.4)	0 (0)	0.601
Other, *n* (%)	67 (15.4)	19 (8.7)	0.443
CSF abnormalities			
Protein >45 mg/dL, *n* (%)	189 (43.3)	49 (47.8)	0.436
WBC >5 cells/μL, *n* (%)	113 (25.9)	54 (52.4)	<0.001
Glucose <50 mg/dL, *n* (%)	33 (7.6)	14 (13.6)	0.051
IgG index >0.66 *n*/*N* (%)	27/356 (7.6)	48/92 (52.2)	<0.001
Neuroaxial MRI			
Abnormal MRI, *n*/*N* (%)	260/412 (63.1)	86/99 (86.9)	<0.001
Length of follow‐up, median [IQR]	24 [1–58]	39 [6–64]	0.102
Mortality, *n* (%)	124 (28.4)	18 (17.5)	0.023

CSF, cerebrospinal fluid; IQR, interquartile range; MRI: magnetic resonance imaging; OCB, oligoclonal bands; SD, standard deviation.

^a^ 2 Uterine cancer, 1 Anaplastic astrocytoma, 1 glioma, 1 cholangiocarcinoma, 1 rectal cancer, 1 tongue cancer, 5 Nonmelanoma skin cancer (2 squamous cell carcinoma, 2 basal cell carcinoma, 1 unspecified).

^b^ 1 leiomyosarcoma, 1 Kaposi sarcoma, 3 Nonmelanoma skin cancer (1 squamous cell carcinoma, 1 basal cell carcinoma, 1 unspecified).

Abnormalities in SPEP were more common in matched‐only, compared to matched + unique patients (55.8% vs 37.5%, *p* = 0.035), though no single SPEP abnormality accounted for this difference (Table 2). Notably, 10% (19/190) of the matched‐only group had an M‐protein but no one in the matched + unique cohort had a positive M‐protein, though this finding was not statistically significant due to the low numbers. Regarding patients with an M protein on SPEP, immunofixation showed a variety of immunoglobulin classes including IgG, immunoglobulin A, and immunoglobulin M, with a mixture of kappa and lambda light chains. Of the 19 patients who had a positive M‐protein in the matched‐only group, 10 had hematologic cancer (7 had multiple myeloma (MM), 1 had marginal zone lymphoma, 1 had follicular lymphoma, and 1 had chronic myelogenous leukemia), 3 had nonhematologic cancer (1 breast cancer, 1 colon cancer, and 1 prostate cancer), and 6 had no history of cancer.

**Table 2:** Serum protein electrophoresis result comparing matched‐only OCB and matched + unique OCB.

	Matched OCB (*n* = 190)	Matched + unique OCB (*n* = 40)	*p*‐value
Abnormal SPEP, *n* (%)	106 (55.8%)	15 (37.5%)	0.035
Decreased albumin	46 (24.2%)	10 (25%)	0.916
M‐protein	19 (10%)^a^	0 (0%)	0.051
Hypogammopathy	20 (10.5%)	2 (5%)	0.383
Other gamma region abnormalities^b^	57 (30%)	9 (22.5%)	0.442

OCB, oligoclonal band; SPEP, serum protein electrophoresis.

^a^ Of the 19 patients, 7 had multiple myeloma, 1 had marginal zone lymphoma, 1 had follicular lymphoma, and 1 chronic myelogenous leukemia.

^b^ Polyclonal increased or restriction of protein migration in the gamma region, or beta‐gamma bridging.

Table 3 shows the final diagnosis of patients in our study. In patients who had matched‐only, the prevalence of NIND was higher compared to matched + unique (68.1% vs 25.2%; *p* < 0.001). Of the 297 patients with NIND, conditions classified as other/not clear (noninflammatory) (*n* = 55/297, 18.5%), stroke (*n* = 48/297, 16.2%), toxic/metabolic conditions (*n* = 48/297, 16.2%), and seizures (*n* = 30/297, 10.1%) together accounted for 60% of the NIND cases. Other‐IND were found in 30% (*N* = 131) of patients with matched bands; Guillain‐Barré syndrome (GBS)/chronic inflammatory demyelinating polyneuropathy (CIDP) (*n* = 33/131, 25.2%), other systemic rheumatologic diseases (*n* = 25/131, 19.1%), and CNS infections (*n* = 24/131, 18.3%) were the most frequent diagnosis accounting for 62.6% of these. Although less common, neuromyelitis optica spectrum disorder (NMOSD) and myelin oligodendrocyte glycoprotein antibody‐associated disease (MOGAD) were only diagnosed in matched‐only.

**Table 3:** Final diagnosis comparing matched‐only OCB and matched + unique OCB.

	Matched (*N* = 436)		Matched + unique (*N* = 103)		*p*‐value
Multiple sclerosis	8	1.8%	34	33%	<0.001
Other inflammatory neurologic disorder					
Autoimmune encephalitis	14	*N* = 131 30%	9	*N* = 43 41.7%	0.022
NMOSD	4		0		
MOGAD	3		0		
CNS infections	24		20		
CNS vasculitis	5		3		
Other noninfectious inflammatory CNS disease	8		3		
GBS/CIDP	33		1		
Bell's palsy secondary to infection	2		1		
Other inflammatory neuropathy	7		2		
Sarcoidosis	6		1		
Other systemic rheumatologic diseases with neurologic involvement	25^a^		3^b^		
Noninflammatory neurologic disorders and other conditions					
Malignancy involving CNS	21	*N* = 297 68.1%	2	*N* = 26 25.2%	<0.001
Stroke	48		3		
PRES	7		1		
RCVS	1		0		
Seizure	30		2		
Toxic/metabolic condition	48		5		
Neurodegenerative condition	10		0		
Noninflammatory neuropathy	12^c^		1^d^		
ALS	4		0		
Primary headache	13		1		
IIH	7		0		
Spondylosis	6		0		
Hypoxic brain injury	3		0		
Malnutritional condition	8		0		
Psychiatric/functional condition	17		0		
Medication side‐effect	5		0		
CJD	2		0		
Other/not clear (noninflammatory)	55		11		

ALS, amyotrophic lateral sclerosis; CJD, Creutzfeldt‐Jacob disease; CNS; central nervous system; GBS/CIDP, Guillain‐Barré syndrome/Chronic inflammatory demyelinating polyneuropathy; IIH, idiopathic intracranial hypertension; MOGAD; myelin oligodendrocyte glycoprotein antibody‐associated disease; NMOSD, neuromyelitis optic spectrum disorder; OCB, oligoclonal band; POTS, postural orthostatic tachycardia syndrome; PRES, posterior reversible encephalopathy syndrome; RA, rheumatoid arthritis; RCVS, reversible cerebral vasoconstriction syndrome; SLE/APLS, systemic lupus erythematosus/anti‐phospholipid syndrome.

^a^ Included 11 SLE/APLS, 5 Sjogren syndrome, 1 RA, 1 Behcet disease, and 7 systemic vasculitis.

^b^ Included 2 SLE/APLS and 1 systemic vasculitis.

^c^ Included 2 idiopathic Bell's palsy and 2 pure autonomic failure/POTS.

^d^ Included 1 idiopathic Bell's palsy.

Matched + unique, compared to matched‐only, had a higher prevalence of MS (33% vs. 1.8%, *p* < 0.001) and other‐IND (41.7% vs. 30%, *p* = 0.022). Of the other‐IND (*N* = 43), CNS infection (*n* = 20/43, 46.5%) was the most frequent diagnosis, followed by autoimmune encephalitis (*n* = 9/43, 20.9%). GBS/CIDP (*n* = 1/43, 2.3%) and other systemic rheumatologic diseases (*n* = 3/43, 7%) were less common in the matched + unique group. Seven sarcoidosis patients were included in our study; six in the matched‐only cohort (all were biopsy proven, all of them had neurological involvement), and 1 in the matched + unique cohort (biopsy proven with neurological involvement). These results remained consistent across groups in patients with CSF RBC <10 cells/μL (Supplement 3). Additionally, the distribution of patient numbers within both matched‐only and matched + unique OCB groups demonstrated consistency across various CSF RBC categories (Supplement 4). This indicates that serum contamination from traumatic taps did not influence these findings.

The subanalysis, conducted on the matched‐only group with available IgG data, is presented in Supplement 5 (*N* = 356). We hypothesized that the IgG index could assist in detecting CNS inflammatory conditions in the matched‐only group. While the proportion of MS was the same in both matched + elevated IgG index and matched + normal IgG index groups, the proportion of other‐IND was higher in the matched + elevated IgG index group compared to the normal IgG index group (55.6% vs. 30.4%, *p* = 0.013). Among the matched‐only cohort with an elevated IgG index, 21 out of 27 (77.8%) had an elevated QAlb, while 128 out of 329 (38.9%) of normal IgG index matched‐only cohort had a high QAlb (*p* < 0.001).

A total of 40 patients (23 matched‐only and 17 match + unique) had ≥2 separate CSF examinations during the study period. Of the matched‐only who had additional CSF analysis, 14 (60.9%) remained matched‐only (4 toxic/metabolic, 3 autoimmune encephalitis, 2 CNS infection, 1 CIDP, 1 systemic vasculitis, 1 stroke, 1 medication side‐effect, and 1 other/not clear), while 9 reverted to negative OCB. Of the 14 patients who remained matched‐only, 2 patients developed 1 unique band in the CSF (both had toxic metabolic encephalopathy; one in the setting of a disseminated tuberculosis infection, and 1 in the setting of tacrolimus toxicity). Of the 17 matched + unique who had additional CSF analysis, 10 (58.8%) remained matched + unique (2 CNS vasculitis, 4 CNS infection, 2 autoimmune encephalitis, 1 systemic vasculitis, and 1 other/noninflammatory condition), while 5 became unique OCB only (1 MS, 1 acute disseminated encephalomyelitis, 1 CNS infection, 1 CNS malignancy, and 1 other/not clear), and 2 became matched‐only.

**Figure 1:**
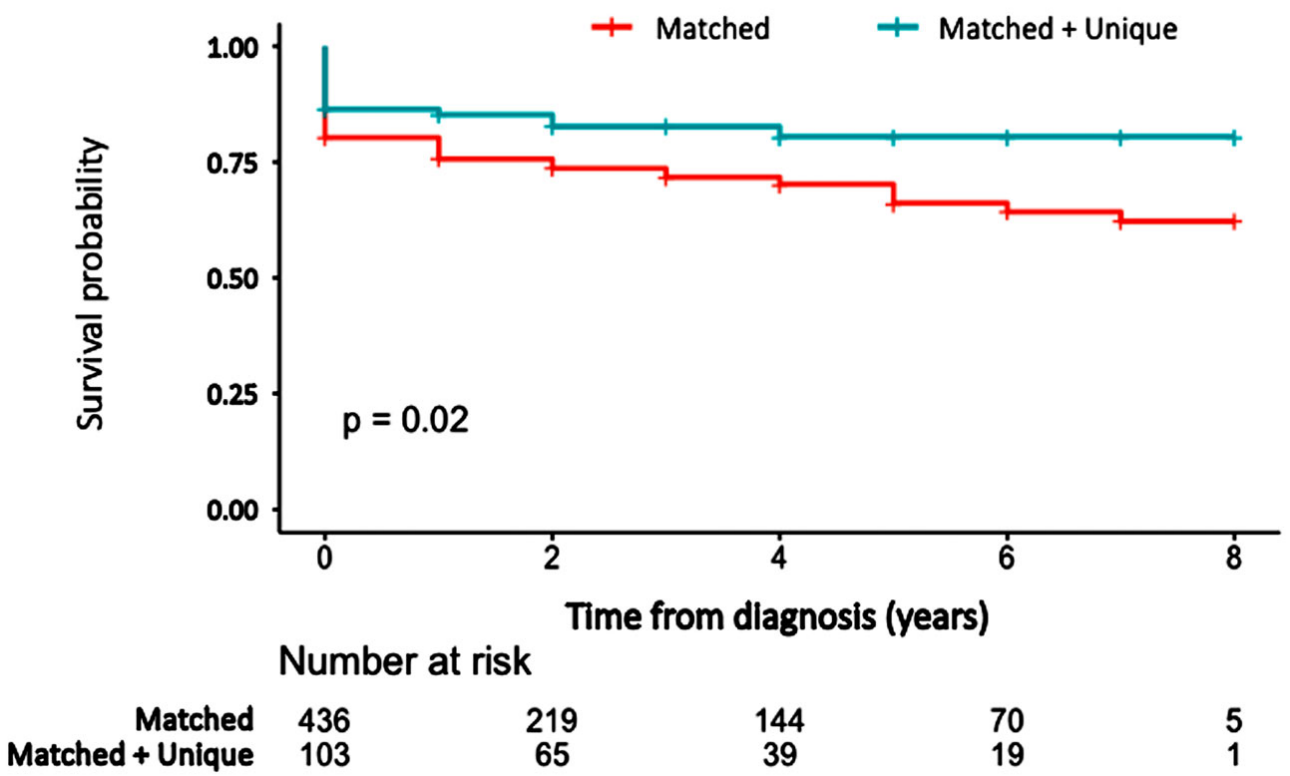
Kaplan–Meier survival estimate based on oligoclonal bands result.

At the time of last follow‐up, 142 patients (26.3%) in the cohort died, including 124 (28.4%) in the matched OCB only cohort and 18 (17.5%) in the matched + unique OCB cohort (*p* = 0.023). The Kaplan–Meier survival curves revealed a significantly higher mortality rate in the matched‐only group compared to the matched + unique group (*p* = 0.02) (Fig. 1). When adjusting for various factors through Cox proportional hazards regression, the difference in mortality attributed to being in the matched‐only OCB group was not statistically significant (Table 4). Analysis of other covariates including age, sex, history of autoimmune disorder, pleocytosis, protein elevation, and low glucose in the CSF was also not associated with mortality in our cohort. Notably, a history of cancer, which was more common in matched‐only, was a significant predictor of increased mortality risk (HR = 3.1472, 95% CI [2.1962, 4.51]).

**Table 4:** Survival estimates comparison in matched‐only OCB and matched + unique OCB groups using Cox proportional hazard model.

	Hazard ratio	95% confidence interval	*p*‐value
Matched‐only OCB	1.4043	0.8363–2.3579	0.199
Age	1.0168	0.9928–1.0415	0.172
Age ≥60 years	0.744	0.3912–1.415	0.367
Age ≥65 years	1.2966	0.7247–2.3197	0.381
Male sex	0.9934	0.6953–1.4194	0.971
History of autoimmune condition	0.9777	0.688–1.3894	0.9
History of cancer	3.1472	2.1962–4.51	< 0.001
CSF white blood cell >5/μL	1.0804	0.7373–1.5831	0.692
CSF protein >50 mg/dL	1.2146	0.8446–1.7467	0.294
CSF glucose <45 mg/dL	1.3452	0.7902–2.2902	0.275

CSF, Cerebrospinal fluid; OCB, oligoclonal band.

## Discussion

Our study details the clinical characteristics and diagnostic utility of matched bands. The matched‐only cohort was older, had a higher prevalence of autoimmune diseases and/or cancer compared to the matched + unique cohort. Over two‐thirds of the patients with matched‐only OCB presented with NIND, while those with matched + unique OCB were more likely to exhibit pleocytosis, elevated IgG synthesis, abnormal MRI findings, and to have been diagnosed with MS or other‐IND.

The diagnoses within the matched‐only cohort who had other‐IND were diverse, including demyelinating disorders such as NMOSD and MOGAD. Both conditions have been reported to present with unique OCB in less than 20% of cases, and their absence in CSF is often more indicative of these conditions.^10^, ^11^ In our cohort, among the seven patients with NMOSD or MOGAD with matched OCB, none exhibited matched + unique bands. This observation highlights the widely accepted view that autoantibodies in NMOSD and MOGAD are primarily produced in the periphery.^12^ It also delineates the unique patterns of neuroinflammation in these conditions, distinguishing them from those seen in MS. Additionally, sarcoidosis, a multisystem disease with granulomatous inflammation, can involve the CNS, where matched bands may offer significant diagnostic value. In a study of 70 neurosarcoidosis patients (10 definite and 60 probable), only two exhibited unique OCB, whereas eight showed matched OCB, and 60 were negative.^13^ Our cohort included seven sarcoidosis cases with neurologic involvement, 6 matched and 1 matched + unique, reinforcing the idea that matched OCB may be a relevant biomarker in inflammatory CNS disorders such as neurologic involvement of sarcoidosis. GBS/CIDP and systemic rheumatologic diseases were well‐represented in the matched‐only group; this result is consistent with the prior studies showing the association of matched‐only OCB and systemic inflammatory conditions and GBS.^4^, ^5^ Stroke and seizure were relatively common in our matched‐only group classified under NIND. Although the connection between stroke or seizure and matched‐only OCB remains under‐explored, some studies have documented the prevalence of unique OCB in these conditions. For instance, prior research indicates that 2.4%–25% of patients with acute ischemic stroke exhibit unique OCB; with the lower 2.4% prevalence noted upon excluding those with chronic inflammatory or rheumatic diseases.^14^, ^15^ In seizure patients, unique OCB are rare (0–8%), and the presence of unique OCB hints at possible autoimmune or infectious etiologies.^16^ The relatively high prevalence of the conditions in matched‐only group classified under NIND could be attributed to the combined effects of systemic autoimmune history and blood–brain barrier dysfunction, which allow greater permeability for systemic immunoglobulins to enter the CSF.

The most common condition associated with matched + unique OCB was MS, seen in almost a third. While most literature on MS focuses on the high prevalence of unique OCB, reflecting an immune response within the CNS, it is important to note that the presence of matched + unique OCB pattern can still support the diagnosis of MS when considered alongside other clinical and radiological findings. Our study further broadens the scope of conditions associated with matched + unique bands. For example, CNS inflammatory conditions such as CNS infections and autoimmune encephalitis were relatively common, whereas inflammatory peripheral neuropathy and systemic rheumatologic diseases were less common. This observed trend is compelling, given that the presence of a unique band is more indicative of CNS inflammation.^2^ Consequently, the identification of matched + unique OCB patterns serves to corroborate diagnostic test outcomes within a clinical setting. A prior study conducted in 1993 compared 56 matched‐only and 46 matched + unique cases; they showed that neoplastic conditions, GBS, and other varieties of peripheral neuropathy were common in the matched‐only group, while MS was the predominant diagnosis in the matched + unique group, similar to our study.^5^ This study predates the discovery of many serologic markers, including aquaporin‐4 IgG and myelin oligodendrocyte glycoprotein IgG, which are important for diagnosing NMOSD and MOGAD, respectively.

The IgG index, a quantitative analysis of the relationship between CSF IgG and serum IgG, divided by the same ratio for albumin, is often elevated in neuroinflammatory disorders, and is well‐established in CNS inflammatory diseases like MS.^17^ However, its significance in cases with matched OCB remains uncertain. Our investigation into the matched‐only OCB subset, distinguishing between normal and high IgG index levels, reveals a marked disparity: individuals with a high IgG index exhibited other‐IND in 55.6% of cases, compared to 30.4% in those with a low IgG index. Previous research has shown that the CSF/serum concentration of IgG (QIgG) is related to the QAlb in a nonlinear manner as exemplified by Reiber's hyperbolic discrimination line.^7^ In our study, the high IgG index group showed a higher proportion of elevated QAlb, suggesting that a higher IgG index may reflect more severe blood‐CSF barrier dysfunction. Moreover, it is important to note that OCB are a more sensitive method for detecting intrathecal IgG synthesis than IgG index in MS.^17^ The added value of the IgG index for detecting CNS inflammatory conditions in conjunction with other biomarkers in the matched‐only cohort requires further investigation.

We observed a relatively high prevalence of history of autoimmune disorder and cancer, particularly in the matched‐only group. These observations align with previous findings on systemic inflammatory responses in various cancer types, including hematologic cancer, breast cancer, and pulmonary cancer.^18^ Although not statistically significant, it is also important to note that hematologic cancers such as lymphoma, leukemia, and MM were more prevalent in the matched‐only group, which also presented with a higher frequency of M‐proteins. In fact, none of the matched + unique patients had MM or M‐protein in our study. The higher prevalence of cancer in the matched‐only group correlated with poorer outcomes on the Kaplan–Meier survival estimate curve. The frequency of cancer could be explained by the older age in the matched‐only group, while the high prevalence of autoimmune disorders in the matched‐only group may be explained by systemic inflammation causing blood–brain barrier leak of OCB.

The dynamic nature of OCB patterns, potentially changing over the course of a disease, presents another layer of complexity. As repeat CSF analysis is infrequently performed, the literature concerning this issue is limited. One study reviewing the dynamics of OCB in patients with various neurologic diseases who had repeated CSF analysis did not reveal practical benefits from repeating CSF analysis for OCB.^19^ We noted changes in OCB status in a few patients during the study period, highlighting the variability in these conditions. None of the matched only developed ≥2 unique OCB during follow‐up (2 patients developed 1 unique OCB), and 88.2% (15 out of 17) of the matched + unique remained positive for ≥2 unique OCB. We could not find a pattern between the diagnosis and changes in repeat OCB testing.

While our study's retrospective nature and single‐center focus may introduce certain biases, its strengths lie in a large sample size and inclusive criteria, providing a comprehensive overview of matched OCB in neurological disorders and highlighting the diagnostic utility of OCB patterns beyond MS. It also reinforces that MS patients have quite frequently matched bands in addition to unique CSF bands. These insights can guide clinicians in interpreting OCB results and directing further diagnostic evaluation. The absence of a control group with no bands (Type 1) is a limitation that should be addressed in future research. In addition, while the IgG index was included as a subanalysis, its sensitivity for detecting intrathecal IgG synthesis is limited.^17^ Future studies incorporating additional markers such as kappa free light chains and kappa index may further elucidate the role of humoral responses in neurologic disease.

Overall, this study demonstrates that patients with matched only versus matched + unique OCB have distinct clinical profiles. Matched‐only patients were older and had a higher frequency of systemic autoimmune conditions. They also had a higher frequency of cancer, which was a significant predictor of increased mortality risk. Nearly one third of patients with matched‐only bands, and over half of patients with matched‐only bands plus an elevated IgG index, have inflammatory neurologic diseases, with diverse diagnoses. Patients with matched + unique bands presented predominantly with inflammatory neurologic disorders, including 33% with MS and 41.7% with other‐IND. When matched bands are found in routine clinical care, the result should be noted rather than disregarded, as matched bands have distinct clinical and diagnostic implications. Future research should include a comparison with patients who have no bands to further support these findings.

## Funding Information

The authors received no financial support for the research and/or publication of this article.

## Conflict of Interest

YH, SJR, JTP, SCL, JEG, JWR, LJ, TLS, and AD have nothing to disclose. JRA: Served on scientific advisory boards for of EMD Serono, Genentech, Horizon; has received research support from Horizon. MMPS: Has received research funding from Biogen, Novartis, Clene Nanomedicine, NIH, National MS Society, Department of Veterans Affairs, and Western Institute for Biomedical Research; has served on an advisory board for TG Therapeutics; and serves on the editorial board for Journal of Neuroimaging. LKP: Consulting/Ad Board: Werfen. SLC: Editor, Neurology®Podcast and Neurology Minute™; Editorial Board N2. Research and/or Clinical Fellowship support from: NIH/NINDS U01NS120901, the Western Institute for Biomedical Research, the SRNA, Alexion, and Barbara Gural Steinmetz Foundation. Consulting/Ad Board: Alexion, Genentech, VielaBio (fees paid to University of Utah Development account).

## Supporting information


Supplementary 1.

Supplementary 2.

Supplementary 3.

Supplementary 4.

Supplementary 5.

## Data Availability

The data that support the findings of this study are available from the corresponding author upon reasonable request.

## References

[1] Thompson AJ , Banwell BL , Barkhof F , et al. Diagnosis of multiple sclerosis: 2017 revisions of the McDonald criteria. Lancet Neurol. 2018;17(2):162‐173. 29275977 10.1016/S1474-4422(17)30470-2

[2] Haertle M , Kallweit U , Weller M , Linnebank M . The presence of oligoclonal IgG bands in human CSF during the course of neurological diseases. J Neurol. 2014;261(3):554‐560. 24449061 10.1007/s00415-013-7234-2

[3] Freedman MS , Thompson EJ , Deisenhammer F , et al. Recommended standard of cerebrospinal fluid analysis in the diagnosis of multiple sclerosis: a consensus statement. Arch Neurol. 2005;62(6):865‐870. 15956157 10.1001/archneur.62.6.865

[4] Thompson EJ . Cerebrospinal fluid. J Neurol Neurosurg Psychiatry. 1995;59(4):349‐357. 7561910 10.1136/jnnp.59.4.349 PMC486067

[5] Zeman A , McLean B , Keir G , Luxton R , Sharief M , Thompson E . The significance of serum oligoclonal bands in neurological diseases. J Neurol Neurosurg Psychiatry. 1993;56(1):32‐35. 8381471 10.1136/jnnp.56.1.32 PMC1014760

[6] Tusseau M , Cheli E , Marignier R , et al. Clinical significance of a single cerebrospinal fluid immunoglobulin band: a retrospective study. Mult Scler. 2021;27(9):1451‐1454. 33295240 10.1177/1352458520978222

[7] Reiber H , Otto M , Trendelenburg C , Wormek A . Reporting cerebrospinal fluid data: knowledge base and interpretation software. Clin Chem Lab Med. 2001;39(4):324‐332. 11388657 10.1515/CCLM.2001.051

[8] Fortini AS , Sanders EL , Weinshenker BG , Katzmann JA . Isoelectric focusing with IgG immunoblotting compared with high‐resolution agarose gel electrophoresis and cerebrospinal fluid IgG index. Am J Clin Pathol. 2003;120(5):672‐675. 14608891 10.1309/EM7K-CQR4-GLMH-RCX4

[9] Teunissen CE , Petzold A , Bennett JL , et al. A consensus protocol for the standardization of cerebrospinal fluid collection and biobanking. Neurology. 2009;73(22):1914‐1922. 19949037 10.1212/WNL.0b013e3181c47cc2 PMC2839806

[10] Jarius S , Pellkofer H , Siebert N , et al. Cerebrospinal fluid findings in patients with myelin oligodendrocyte glycoprotein (MOG) antibodies. Part 1: results from 163 lumbar punctures in 100 adult patients. J Neuroinflammation. 2020;17(1):261. 32883348 10.1186/s12974-020-01824-2 PMC7470615

[11] Wingerchuk DM , Banwell B , Bennett JL , et al. International consensus diagnostic criteria for neuromyelitis optica spectrum disorders. Neurology. 2015;85(2):177‐189. 26092914 10.1212/WNL.0000000000001729 PMC4515040

[12] Akaishi T , Takahashi T , Misu T , et al. Difference in the source of anti‐AQP4‐IgG and anti‐MOG‐IgG antibodies in CSF in patients with Neuromyelitis Optica Spectrum disorder. Neurology. 2021;97(1):e1‐e12. 33980704 10.1212/WNL.0000000000012175 PMC8312856

[13] Arun T , Pattison L , Palace J . Distinguishing neurosarcoidosis from multiple sclerosis based on CSF analysis: a retrospective study. Neurology. 2020;94(24):e2545‐e2554. 32354749 10.1212/WNL.0000000000009491

[14] Prüss H , Iggena D , Baldinger T , et al. Evidence of intrathecal immunoglobulin synthesis in stroke: a cohort study. Arch Neurol. 2012;69(6):714‐717. 22371852 10.1001/archneurol.2011.3252

[15] Laichinger K , Bombach P , Dünschede J , et al. No evidence of oligoclonal bands, intrathecal immunoglobulin synthesis and B cell recruitment in acute ischemic stroke. PLoS One. 2023;18(3):e0283476. 37000850 10.1371/journal.pone.0283476 PMC10065233

[16] Langenbruch L , Wiendl H , Groß C , Kovac S . Diagnostic utility of cerebrospinal fluid (CSF) findings in seizures and epilepsy with and without autoimmune‐associated disease. Seizure. 2021;91:233‐243. 34233238 10.1016/j.seizure.2021.06.030

[17] Link H , Huang YM . Oligoclonal bands in multiple sclerosis cerebrospinal fluid: an update on methodology and clinical usefulness. J Neuroimmunol. 2006;180(1–2):17‐28. 16945427 10.1016/j.jneuroim.2006.07.006

[18] Dolan RD , McMillan DC . The prevalence of cancer associated systemic inflammation: implications of prognostic studies using the Glasgow Prognostic Score. Crit Rev Oncol Hematol. 2020;150:102962. 32344318 10.1016/j.critrevonc.2020.102962

[19] Mermelstein M , Naftali J , Wilf‐Yarkoni A , Lotan I , Hellmann MA , Steiner I . Repeated lumbar puncture in search of oligoclonal bands—what is the yield? J Neurol Sci. 2022;439:120298. 35662071 10.1016/j.jns.2022.120298

