# Supplementary material for: Matched oligoclonal bands: Diagnostic utility and clinical characteristics
Source: Ann Clin Transl Neurol. 2024 Oct 22;11(11):2846–54. doi: 10.1002/acn3.52162 (PMC11572730; doi:10.1002/acn3.52162)
Supplement: Supplementary file 1 — Supplementary 1. [file ACN3-11-2846-s002.docx]

**Supplement 1: Study classification based on previous consensus definitions^6^ with relevant examples.**

| **Multiple sclerosis** | **Other inflammatory neurologic disorder** | **Non-inflammatory neurological disorders and other conditions** |
| --- | --- | --- |
|  | Autoimmune encephalitis | Malignancy involving CNS |
|  | NMOSD | Stroke |
|  | MOGAD | PRES |
|  | CNS infections | RCVS |
|  | CNS vasculitis | Seizure |
|  | Other non-infectious Inflammatory CNS disease^a^ | Toxic/metabolic condition |
|  | GBS/CIDP | Neurodegenerative condition |
|  | Bell’s palsy secondary to infection | Non-inflammatory neuropathy |
|  | Other inflammatory neuropathy^b^ | ALS |
|  | Sarcoidosis | Primary headache |
|  | Other systemic rheumatologic diseases with neurological involvement^c^ | IIH |
|  |  | Spondylosis |
|  |  | Hypoxic brain injury |
|  |  | Malnutritional condition |
|  |  | Psychiatric/Functional condition |
|  |  | Medication side-effect |
|  |  | CJD |
|  |  | Other/not clear (non-inflammatory) |

ALS, amyotrophic lateral sclerosis; CJD, Creutzfeldt-Jacob disease; CNS; central nervous system; GBS/CIDP, Guillain-Barré syndrome/Chronic inflammatory demyelinating polyneuropathy; IIH, idiopathic intracranial hypertension; MOGAD; myelin oligodendrocyte glycoprotein antibody-associated disease; NMOSD, neuromyelitis optic spectrum disorder; PRES, posterior reversible encephalopathy syndrome; RCVS, reversible cerebral vasoconstriction syndrome

^a^Includes medication induced aseptic meningitis, inflammatory cerebral amyloid angiopathy, Cogan syndrome, Susac’s syndrome, idiopathic transverse myelitis

^b^Includes autoimmune autonomic ganglionopathy, POEMS syndrome, inflammatory neuropathy of unclear etiology

^c^Includes Systemic lupus erythematosus/anti-phospholipid syndrome, Sjogren syndrome, rheumatoid arthritis, Behcet disease, and systemic vasculitis
