# Supplementary material for: Matched oligoclonal bands: Diagnostic utility and clinical characteristics
Source: Ann Clin Transl Neurol. 2024 Oct 22;11(11):2846–54. doi: 10.1002/acn3.52162 (PMC11572730; doi:10.1002/acn3.52162)
Supplement: Supplementary file 2 — Supplementary 2. [file ACN3-11-2846-s003.docx]

**Supplement 2: Study flow chart of the derivation of the analytic samples.**
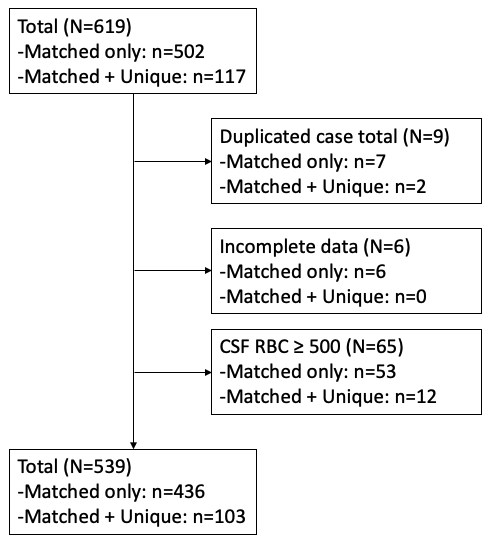


CSF, cerebrospinal fluid; RBC, red blood cell
