# Supplementary material for: Matched oligoclonal bands: Diagnostic utility and clinical characteristics
Source: Ann Clin Transl Neurol. 2024 Oct 22;11(11):2846–54. doi: 10.1002/acn3.52162 (PMC11572730; doi:10.1002/acn3.52162)
Supplement: Supplementary file 3 — Supplementary 3. [file ACN3-11-2846-s004.docx]

**Supplement 3:** **Final diagnosis comparing matched-only OCB and matched + unique OCB (CSF RBC <10).**

| **Conditions** | | **Matched**  **(N=280)** | | **Matched + Unique (N=65)** | | **P-value** |
| --- | --- | --- | --- | --- | --- | --- |
| **Multiple sclerosis** | | 6 | 2.1% | 22 | 33.8% | <0.001 |
| **Other inflammatory neurologic disorder** | Autoimmune encephalitis | 9 | N=87 31.1% | 6 | N=29 44.6% | 0.037 |
|  | NMOSD | 2 |  | 0 |  |  |
|  | MOGAD | 2 |  | 0 |  |  |
|  | CNS infections | 15 |  | 13 |  |  |
|  | CNS vasculitis | 3 |  | 1 |  |  |
|  | Other non-infectious Inflammatory CNS disease | 7 |  | 2 |  |  |
|  | GBS/CIDP | 23 |  | 1 |  |  |
|  | Bell’s palsy secondary to infection | 1 |  | 1 |  |  |
|  | Other inflammatory neuropathy | 2 |  | 2 |  |  |
|  | Sarcoidosis | 3 |  | 1 |  |  |
|  | Other systemic rheumatologic diseases with neurological involvement | 23^a^ |  | 2^b^ |  |  |
| **Non-inflammatory neurological disorders and other conditions** | Malignancy involving CNS | 16 | N=187  66.8% | 1 | N=14 21.5% | <0.001 |
|  | Stroke | 29 |  | 1 |  |  |
|  | PRES | 5 |  | 1 |  |  |
|  | RCVS | 0 |  | 0 |  |  |
|  | Seizure | 14 |  | 0 |  |  |
|  | Toxic/metabolic condition | 26 |  | 3 |  |  |
|  | Neurodegenerative condition | 5 |  | 0 |  |  |
|  | Non-inflammatory neuropathy | 5^c^ |  | 1^d^ |  |  |
|  | ALS | 2 |  | 0 |  |  |
|  | Primary headache | 11 |  | 1 |  |  |
|  | IIH | 7 |  | 0 |  |  |
|  | Spondylosis | 4 |  | 0 |  |  |
|  | Hypoxic brain injury | 1 |  | 0 |  |  |
|  | Malnutritional condition | 5 |  | 0 |  |  |
|  | Psychiatric/Functional condition | 10 |  | 0 |  |  |
|  | Medication side-effect | 3 |  | 0 |  |  |
|  | CJD | 2 |  | 0 |  |  |
|  | Other/not clear (non-inflammatory) | 42 |  | 6 |  |  |

ALS, amyotrophic lateral sclerosis; CJD, Creutzfeldt-Jacob disease; CNS; central nervous system; GBS/CIDP, Guillain-Barré syndrome/Chronic inflammatory demyelinating polyneuropathy; IIH, idiopathic intracranial hypertension; MOGAD; myelin oligodendrocyte glycoprotein antibody-associated disease; NMOSD, neuromyelitis optic spectrum disorder; OCB, oligoclonal band; POTS, postural orthostatic tachycardia syndrome; PRES, posterior reversible encephalopathy syndrome; RA, rheumatoid arthritis; RCVS, reversible cerebral vasoconstriction syndrome; SLE/APLS, systemic lupus erythematosus/anti-phospholipid syndrome

^a^Included 7 SLE/APLS, 5 Sjogren syndrome, 1 RA, and 7 systemic vasculitis.
^b^Included 1 SLE/APLS and 1 systemic vasculitis.

^c^Included 1 pure autonomic failure/POTS.

^d^Included 1 idiopathic Bell’s palsy.
