# Supplementary material for: Matched oligoclonal bands: Diagnostic utility and clinical characteristics
Source: Ann Clin Transl Neurol. 2024 Oct 22;11(11):2846–54. doi: 10.1002/acn3.52162 (PMC11572730; doi:10.1002/acn3.52162)
Supplement: Supplementary file 4 — Supplementary 4. [file ACN3-11-2846-s001.docx]

**Supplement 4: Distribution of matched-only and matched + unique OCB in Different CSF RBC categories.**

|  | **Matched** | **Matched + Unique** | **Total** |
| --- | --- | --- | --- |
| **RBC < 500** | 436 (80.9%) | 103 (19.1%) | 539 |
| **RBC < 100** | 384 (81.2%) | 89 (18.8%) | 473 |
| **RBC < 10** | 280 (81.2%) | 65 (18.8%) | 345 |

CSF, cerebrospinal fluid; OCB, oligoclonal band; RBC, red blood cell
