# Supplementary material for: Matched oligoclonal bands: Diagnostic utility and clinical characteristics
Source: Ann Clin Transl Neurol. 2024 Oct 22;11(11):2846–54. doi: 10.1002/acn3.52162 (PMC11572730; doi:10.1002/acn3.52162)
Supplement: Supplementary file 5 — Supplementary 5. [file ACN3-11-2846-s005.docx]

**Supplement 5:** **Final diagnosis comparing matched-only OCB with IgG index ≤0.66 and matched-only OCB with IgG index >0.66.**

| **Conditions** | | **Matched (IgG index≤0.66)**  **(N=329)** | | **Matched**  **(IgG index>0.66) (N=27)** | | **P-value** |
| --- | --- | --- | --- | --- | --- | --- |
| **Multiple sclerosis** | | 8 | 2.4% | 0 | 0% | 0.885 |
| **Other inflammatory neurologic disorder** | Autoimmune encephalitis | 11 | N=100  30.4% | 2 | N=15 55.6% | 0.013 |
|  | NMOSD | 2 |  | 2 |  |  |
|  | MOGAD | 3 |  | 0 |  |  |
|  | CNS infections | 20 |  | 2 |  |  |
|  | CNS vasculitis | 5 |  | 0 |  |  |
|  | Other non-infectious Inflammatory CNS disease | 8 |  | 0 |  |  |
|  | GBS/CIDP | 21 |  | 4 |  |  |
|  | Bell’s palsy secondary to infection | 2 |  | 0 |  |  |
|  | Other inflammatory neuropathy | 6 |  | 0 |  |  |
|  | Sarcoidosis | 5 |  | 1 |  |  |
|  | Systemic rheumatologic diseases with neurological involvement | 17^a^ |  | 4^b^ |  |  |
| **Non-inflammatory neurological disorders and other conditions** | Malignancy involving CNS | 17 | N=221  67.2% | 0 | N=12 44.4% | 0.03 |
|  | Stroke | 31 |  | 3 |  |  |
|  | PRES | 6 |  | 0 |  |  |
|  | RCVS | 1 |  | 0 |  |  |
|  | Seizure | 25 |  | 2 |  |  |
|  | Toxic/metabolic condition | 29 |  | 4 |  |  |
|  | Neurodegenerative condition | 9 |  | 0 |  |  |
|  | Non-inflammatory neuropathy | 8^c^ |  | 0 |  |  |
|  | ALS | 4 |  | 0 |  |  |
|  | Primary headache | 8 |  | 2 |  |  |
|  | IIH | 7 |  | 0 |  |  |
|  | Spondylosis | 6 |  | 0 |  |  |
|  | Hypoxic brain injury | 2 |  | 0 |  |  |
|  | Malnutritional condition | 8 |  | 0 |  |  |
|  | Psychiatric/Functional condition | 13 |  | 0 |  |  |
|  | Medication side-effect | 4 |  | 0 |  |  |
|  | CJD | 2 |  | 0 |  |  |
|  | Other/not clear (non-inflammatory) | 41 |  | 1 |  |  |

ALS, amyotrophic lateral sclerosis; CJD, Creutzfeldt-Jacob disease; CNS; central nervous system; GBS/CIDP, Guillain-Barré syndrome/Chronic inflammatory demyelinating polyneuropathy; IIH, idiopathic intracranial hypertension; MOGAD; myelin oligodendrocyte glycoprotein antibody-associated disease; NMOSD, neuromyelitis optic spectrum disorder; OCB, oligoclonal band; POTS, postural orthostatic tachycardia syndrome; PRES, posterior reversible encephalopathy syndrome; RA, rheumatoid arthritis; RCVS, reversible cerebral vasoconstriction syndrome; SLE/APLS, systemic lupus erythematosus/anti-phospholipid syndrome

^a^Included 6 SLE/APLS, 5 Sjogren syndrome, and 6 systemic vasculitis.
^b^Included 4 SLE/APLS.

^c^Included 1 idiopathic Bell’s palsy and 1 pure autonomic failure/POTS.
